# (*E*)-2-(4-Diethyl­amino-2-hydroxy­benzyl­idene­amino)benzonitrile

**DOI:** 10.1107/S160053681000111X

**Published:** 2010-02-06

**Authors:** Xing-Cong Wang, Hua Xu, Kun Qian

**Affiliations:** a Jiangxi Key Laboratory of Organic Chemistry, Jiangxi Science and Technology Normal University, Nanchang 330013, People’s Republic of China; b International Education College of Jiangxi University of Traditional Chinese Medicine, NanChang 330004, People’s Republic of China; c Academic Administration of Jiangxi University of Traditional Chinese Medicine, NanChang 330004, People’s Republic of China

## Abstract

The mol­ecule of the title compound, C_18_H_19_N_3_O, displays a *trans* configuration with respect to the C=N double bond. The dihedral angle between the planes of the two benzene rings is 2.62 (11)°. A strong intra­molecular O—H⋯N hydrogen bond stabilizes the mol­ecular conformation.

## Related literature

For the properties of Schiff bases compounds, see: Weber *et al.* (2007 ▶). Chen *et al.* (2008 ▶). May *et al.* (2004 ▶).
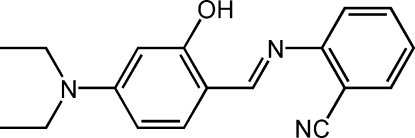


## Experimental

### 

#### Crystal data

C_18_H_19_N_3_O
*M* *_r_* = 293.36
Monoclinic, 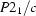

*a* = 7.185 (5) Å
*b* = 12.324 (9) Å
*c* = 18.490 (12) Å
β = 108.39 (2)°
*V* = 1553.6 (19) Å^3^
*Z* = 4
Mo *K*α radiation
μ = 0.08 mm^−1^
*T* = 293 K
0.20 × 0.20 × 0.20 mm

#### Data collection

Rigaku SCXmini diffractometer
Absorption correction: multi-scan (*CrystalClear*; Rigaku, 2005 ▶) *T*_min_ = 0.984, *T*_max_ = 0.984
16730 measured reflections
3546 independent reflections
2694 reflections with *I* > 2σ(*I*)
*R*_int_ = 0.062

#### Refinement

*R*[*F* ^2^ > 2σ(*F* ^2^)] = 0.072
*wR*(*F* ^2^) = 0.152
*S* = 1.16
3546 reflections
203 parameters
H atoms treated by a mixture of independent and constrained refinement
Δρ_max_ = 0.22 e Å^−3^
Δρ_min_ = −0.20 e Å^−3^

### 

Data collection: *CrystalClear* (Rigaku, 2005 ▶); cell refinement: *CrystalClear*; data reduction: *CrystalClear*; program(s) used to solve structure: *SHELXS97* (Sheldrick, 2008 ▶); program(s) used to refine structure: *SHELXL97* (Sheldrick, 2008 ▶); molecular graphics: *SHELXTL* (Sheldrick, 2008 ▶); software used to prepare material for publication: *SHELXL97*.

## Supplementary Material

Crystal structure: contains datablocks I, global. DOI: 10.1107/S160053681000111X/bx2261sup1.cif

Structure factors: contains datablocks I. DOI: 10.1107/S160053681000111X/bx2261Isup2.hkl

Additional supplementary materials:  crystallographic information; 3D view; checkCIF report

## Figures and Tables

**Table 1:** Hydrogen-bond geometry (Å, °)

*D*—H⋯*A*	*D*—H	H⋯*A*	*D*⋯*A*	*D*—H⋯*A*
O1—H1*A*⋯N2	0.88 (3)	1.83 (3)	2.623 (3)	149 (3)

**Fig. 1.**
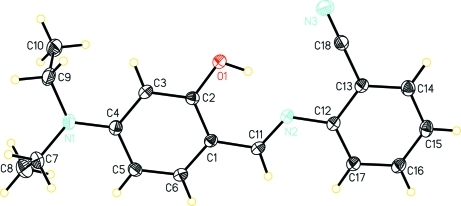
A view of the title compound with the atomic numbering scheme. Displacement ellipsoids are drawn at the 30% probability level.

## supplementary crystallographic information

### Comment

Schiff bases compounds are of great interest in many fields of chemistry and biochemistry, primarily due to their importance in the development of coordination chemistry related to magnetism (Weber, *et al.*, 2007), catalysis (Chen, *et al.*, 2008) and biological process (May, *et al.*,2004). Here, we report the synthesis and crystal structure of the title compound, (I).

Fig. 1 shows *ORTEP* plots of the title compounds. All the bond lengths and angles in the molecules are in the range of normal values. The molecule displays a *trans* configuration about the central C11=N2 bond and adopts the phenol-imine tautomeric form, with a strong intramolecular O—H···N hydrogen bonding interaction (Table 1). The dihedral angle between the mean planes of the two aromatic rings is 2.62 (11) ° indicating that the Schiff-base ligand adopts a coplanar conformation. In addition, two methyl groups are oriented to the same direction relative to the plane of the adjacent benzene ring. The crystal packing is stabilized by van der Waals interactions.

### Experimental

4-aminobenzonitrile (0.590 g, 5 mmol)and 4-(diethylamino)-2-hydroxybenzaldehyde (0.996 g, 5 mmol) were dissolved in ethanol (20 ml). The reaction mixture was stirred for 6 h under reflux, and then cooled to room temperature slowly. The resulting yellow precipitate was filtered off and the yellow crystals of the title compound suitable for X-ray analysis were obtained from acetonitrile solution by slow evaporation.

### Refinement

H atoms (for OH) were located in a difference Fourier map and refined isotropically. The remailing H atoms were located geometrically and treated as riding atoms with C—H = 0.93–0.97 Å, and with *U*_iso_(H) = 1.2 *U*_eq_(C) for aromatic H atoms or 1.5 *U*_eq_ (C) for methyl H atoms.

### Crystal data

**Table:**

C_18_H_19_N_3_O	*F*(000) = 624
*M**_r_* = 293.36	*D*_x_ = 1.254 Mg m^−^^3^
Monoclinic, *P*2_1_/*c*	Mo *K*α radiation, λ = 0.71073 Å
Hall symbol: -P 2ybc	Cell parameters from 3397 reflections
*a* = 7.185 (5) Å	θ = 2.3–27.6°
*b* = 12.324 (9) Å	µ = 0.08 mm^−^^1^
*c* = 18.490 (12) Å	*T* = 293 K
β = 108.39 (2)°	Prism, yellow
*V* = 1553.6 (19) Å^3^	0.20 × 0.20 × 0.20 mm
*Z* = 4	

### Data collection

**Table:**

Rigaku SCXmini diffractometer	3546 independent reflections
Radiation source: fine-focus sealed tube	2694 reflections with *I* > 2σ(*I*)
graphite	*R*_int_ = 0.062
Detector resolution: 13.6612 pixels mm^-1^	θ_max_ = 27.4°, θ_min_ = 2.9°
ω scans	*h* = −9→9
Absorption correction: multi-scan (*CrystalClear*; Rigaku, 2005)	*k* = −15→15
*T*_min_ = 0.984, *T*_max_ = 0.984	*l* = −23→23
16730 measured reflections	

### Refinement

**Table:**

Refinement on *F*^2^	Primary atom site location: structure-invariant direct methods
Least-squares matrix: full	Secondary atom site location: difference Fourier map
*R*[*F*^2^ > 2σ(*F*^2^)] = 0.072	Hydrogen site location: inferred from neighbouring sites
*wR*(*F*^2^) = 0.152	H atoms treated by a mixture of independent and constrained refinement
*S* = 1.16	*w* = 1/[σ^2^(*F*_o_^2^) + (0.0521*P*)^2^ + 0.4099*P*] where *P* = (*F*_o_^2^ + 2*F*_c_^2^)/3
3546 reflections	(Δ/σ)_max_ < 0.001
203 parameters	Δρ_max_ = 0.22 e Å^−^^3^
0 restraints	Δρ_min_ = −0.20 e Å^−^^3^

### Special details

**Table:**

Geometry. All e.s.d.'s (except the e.s.d. in the dihedral angle between two l.s. planes) are estimated using the full covariance matrix. The cell e.s.d.'s are taken into account individually in the estimation of e.s.d.'s in distances, angles and torsion angles; correlations between e.s.d.'s in cell parameters are only used when they are defined by crystal symmetry. An approximate (isotropic) treatment of cell e.s.d.'s is used for estimating e.s.d.'s involving l.s. planes.
Refinement. Refinement of *F*^2^ against ALL reflections. The weighted *R*-factor *wR* and goodness of fit *S* are based on *F*^2^, conventional *R*-factors *R* are based on *F*, with *F* set to zero for negative *F*^2^. The threshold expression of *F*^2^ > σ(*F*^2^) is used only for calculating *R*-factors(gt) *etc*. and is not relevant to the choice of reflections for refinement. *R*-factors based on *F*^2^ are statistically about twice as large as those based on *F*, and *R*- factors based on ALL data will be even larger.

### Fractional atomic coordinates and isotropic or equivalent isotropic displacement parameters (Å^2^)

**Table:**

	*x*	*y*	*z*	*U*_iso_*/*U*_eq_	
O1	−0.0254 (2)	0.40112 (14)	0.61866 (9)	0.0379 (4)	
N1	0.4621 (3)	0.58450 (14)	0.81676 (10)	0.0349 (4)	
N2	0.0535 (2)	0.35141 (13)	0.49350 (9)	0.0269 (4)	
N3	−0.4028 (3)	0.26723 (17)	0.48643 (11)	0.0424 (5)	
C1	0.2850 (3)	0.43963 (16)	0.59876 (11)	0.0255 (4)	
C2	0.1595 (3)	0.44183 (16)	0.64441 (11)	0.0267 (4)	
C3	0.2210 (3)	0.48662 (17)	0.71679 (11)	0.0292 (5)	
H3A	0.1370	0.4851	0.7461	0.035*	
C4	0.4072 (3)	0.53439 (16)	0.74698 (11)	0.0289 (5)	
C5	0.5361 (3)	0.52898 (17)	0.70227 (12)	0.0305 (5)	
H5A	0.6625	0.5568	0.7214	0.037*	
C6	0.4742 (3)	0.48286 (16)	0.63105 (11)	0.0290 (5)	
H6A	0.5612	0.4801	0.6030	0.035*	
C7	0.6397 (4)	0.6504 (2)	0.84374 (14)	0.0460 (6)	
H7A	0.6146	0.7105	0.8733	0.055*	
H7B	0.6681	0.6806	0.7999	0.055*	
C8	0.8184 (4)	0.5898 (3)	0.89198 (16)	0.0615 (8)	
H8A	0.9280	0.6386	0.9078	0.092*	
H8B	0.8478	0.5317	0.8627	0.092*	
H8C	0.7930	0.5606	0.9361	0.092*	
C9	0.3349 (4)	0.58173 (19)	0.86478 (13)	0.0414 (6)	
H9A	0.2019	0.5989	0.8338	0.050*	
H9B	0.3772	0.6373	0.9037	0.050*	
C10	0.3350 (4)	0.4732 (2)	0.90292 (13)	0.0492 (7)	
H10A	0.2488	0.4762	0.9334	0.074*	
H10B	0.4656	0.4565	0.9349	0.074*	
H10C	0.2908	0.4179	0.8647	0.074*	
C11	0.2253 (3)	0.39466 (16)	0.52344 (11)	0.0265 (4)	
H11A	0.3120	0.3965	0.4953	0.032*	
C12	−0.0049 (3)	0.30557 (15)	0.41992 (11)	0.0255 (4)	
C13	−0.1936 (3)	0.25907 (16)	0.39549 (11)	0.0274 (4)	
C14	−0.2688 (3)	0.21019 (17)	0.32369 (12)	0.0335 (5)	
H14A	−0.3934	0.1796	0.3088	0.040*	
C15	−0.1567 (3)	0.20773 (17)	0.27517 (12)	0.0350 (5)	
H15A	−0.2049	0.1754	0.2274	0.042*	
C16	0.0289 (3)	0.25409 (17)	0.29866 (12)	0.0338 (5)	
H16A	0.1037	0.2531	0.2658	0.041*	
C17	0.1058 (3)	0.30176 (17)	0.36960 (11)	0.0317 (5)	
H17A	0.2312	0.3314	0.3840	0.038*	
C18	−0.3098 (3)	0.26378 (17)	0.44614 (12)	0.0319 (5)	
H1A	−0.044 (4)	0.378 (2)	0.5719 (17)	0.071 (9)*	

### Atomic displacement parameters (Å^2^)

**Table:**

	*U*^11^	*U*^22^	*U*^33^	*U*^12^	*U*^13^	*U*^23^
O1	0.0314 (8)	0.0568 (11)	0.0272 (8)	−0.0084 (7)	0.0117 (7)	−0.0068 (8)
N1	0.0439 (11)	0.0333 (10)	0.0276 (9)	−0.0074 (8)	0.0116 (8)	−0.0066 (8)
N2	0.0304 (9)	0.0274 (9)	0.0234 (8)	0.0018 (7)	0.0091 (7)	0.0008 (7)
N3	0.0390 (11)	0.0513 (13)	0.0398 (11)	0.0015 (9)	0.0168 (9)	0.0065 (9)
C1	0.0295 (11)	0.0240 (10)	0.0239 (10)	0.0009 (8)	0.0096 (9)	0.0030 (8)
C2	0.0259 (10)	0.0274 (11)	0.0263 (10)	0.0005 (8)	0.0078 (9)	0.0029 (8)
C3	0.0331 (11)	0.0320 (12)	0.0248 (10)	0.0018 (9)	0.0125 (9)	0.0020 (8)
C4	0.0369 (12)	0.0231 (11)	0.0260 (10)	−0.0001 (9)	0.0091 (9)	0.0009 (8)
C5	0.0288 (11)	0.0310 (11)	0.0301 (11)	−0.0037 (9)	0.0069 (9)	0.0014 (9)
C6	0.0311 (11)	0.0291 (11)	0.0292 (10)	0.0006 (9)	0.0133 (9)	0.0028 (8)
C7	0.0565 (16)	0.0399 (14)	0.0440 (14)	−0.0184 (12)	0.0191 (12)	−0.0163 (11)
C8	0.0448 (16)	0.081 (2)	0.0523 (17)	−0.0146 (15)	0.0062 (13)	−0.0122 (15)
C9	0.0550 (15)	0.0426 (14)	0.0287 (11)	−0.0043 (11)	0.0162 (11)	−0.0083 (10)
C10	0.0609 (17)	0.0565 (17)	0.0289 (12)	−0.0107 (13)	0.0121 (12)	0.0017 (11)
C11	0.0309 (11)	0.0260 (11)	0.0254 (10)	0.0042 (8)	0.0128 (9)	0.0037 (8)
C12	0.0312 (11)	0.0214 (10)	0.0246 (10)	0.0044 (8)	0.0098 (9)	0.0023 (8)
C13	0.0330 (11)	0.0233 (10)	0.0266 (10)	0.0014 (8)	0.0104 (9)	0.0021 (8)
C14	0.0376 (12)	0.0295 (12)	0.0314 (11)	−0.0030 (9)	0.0079 (10)	−0.0014 (9)
C15	0.0461 (13)	0.0280 (12)	0.0287 (11)	−0.0005 (9)	0.0089 (10)	−0.0061 (9)
C16	0.0418 (13)	0.0329 (12)	0.0303 (11)	0.0018 (10)	0.0166 (10)	−0.0025 (9)
C17	0.0321 (11)	0.0347 (12)	0.0294 (11)	0.0000 (9)	0.0111 (9)	−0.0008 (9)
C18	0.0325 (11)	0.0309 (12)	0.0303 (11)	−0.0025 (9)	0.0071 (10)	0.0019 (9)

### Geometric parameters (Å, °)

**Table:**

O1—C2	1.358 (2)	C8—H8A	0.9600
O1—H1A	0.88 (3)	C8—H8B	0.9600
N1—C4	1.371 (3)	C8—H8C	0.9600
N1—C7	1.461 (3)	C9—C10	1.512 (3)
N1—C9	1.462 (3)	C9—H9A	0.9700
N2—C11	1.297 (3)	C9—H9B	0.9700
N2—C12	1.409 (3)	C10—H10A	0.9600
N3—C18	1.148 (3)	C10—H10B	0.9600
C1—C6	1.406 (3)	C10—H10C	0.9600
C1—C2	1.416 (3)	C11—H11A	0.9300
C1—C11	1.433 (3)	C12—C17	1.403 (3)
C2—C3	1.385 (3)	C12—C13	1.409 (3)
C3—C4	1.406 (3)	C13—C14	1.402 (3)
C3—H3A	0.9300	C13—C18	1.439 (3)
C4—C5	1.424 (3)	C14—C15	1.382 (3)
C5—C6	1.373 (3)	C14—H14A	0.9300
C5—H5A	0.9300	C15—C16	1.388 (3)
C6—H6A	0.9300	C15—H15A	0.9300
C7—C8	1.509 (4)	C16—C17	1.383 (3)
C7—H7A	0.9700	C16—H16A	0.9300
C7—H7B	0.9700	C17—H17A	0.9300
			
C2—O1—H1A	107.1 (19)	N1—C9—C10	113.3 (2)
C4—N1—C7	122.16 (19)	N1—C9—H9A	108.9
C4—N1—C9	120.78 (19)	C10—C9—H9A	108.9
C7—N1—C9	116.85 (18)	N1—C9—H9B	108.9
C11—N2—C12	122.03 (17)	C10—C9—H9B	108.9
C6—C1—C2	116.85 (18)	H9A—C9—H9B	107.7
C6—C1—C11	120.79 (18)	C9—C10—H10A	109.5
C2—C1—C11	122.35 (19)	C9—C10—H10B	109.5
O1—C2—C3	117.68 (18)	H10A—C10—H10B	109.5
O1—C2—C1	121.33 (18)	C9—C10—H10C	109.5
C3—C2—C1	120.99 (19)	H10A—C10—H10C	109.5
C2—C3—C4	121.58 (19)	H10B—C10—H10C	109.5
C2—C3—H3A	119.2	N2—C11—C1	121.77 (18)
C4—C3—H3A	119.2	N2—C11—H11A	119.1
N1—C4—C3	121.13 (19)	C1—C11—H11A	119.1
N1—C4—C5	121.4 (2)	C17—C12—C13	117.61 (19)
C3—C4—C5	117.49 (19)	C17—C12—N2	126.53 (19)
C6—C5—C4	120.2 (2)	C13—C12—N2	115.86 (17)
C6—C5—H5A	119.9	C14—C13—C12	121.52 (19)
C4—C5—H5A	119.9	C14—C13—C18	120.3 (2)
C5—C6—C1	122.75 (19)	C12—C13—C18	118.17 (18)
C5—C6—H6A	118.6	C15—C14—C13	119.7 (2)
C1—C6—H6A	118.6	C15—C14—H14A	120.2
N1—C7—C8	114.6 (2)	C13—C14—H14A	120.2
N1—C7—H7A	108.6	C14—C15—C16	119.1 (2)
C8—C7—H7A	108.6	C14—C15—H15A	120.4
N1—C7—H7B	108.6	C16—C15—H15A	120.4
C8—C7—H7B	108.6	C17—C16—C15	121.9 (2)
H7A—C7—H7B	107.6	C17—C16—H16A	119.1
C7—C8—H8A	109.5	C15—C16—H16A	119.1
C7—C8—H8B	109.5	C16—C17—C12	120.2 (2)
H8A—C8—H8B	109.5	C16—C17—H17A	119.9
C7—C8—H8C	109.5	C12—C17—H17A	119.9
H8A—C8—H8C	109.5	N3—C18—C13	179.8 (3)
H8B—C8—H8C	109.5		

### Hydrogen-bond geometry (Å, °)

**Table:**

*D*—H···*A*	*D*—H	H···*A*	*D*···*A*	*D*—H···*A*
O1—H1A···N2	0.88 (3)	1.83 (3)	2.623 (3)	149 (3)
